# Overlaps and differences in the core symptoms of patients with attention-deficit/hyperactivity disorder and patients with borderline personality disorder

**DOI:** 10.3389/fpsyt.2026.1822142

**Published:** 2026-06-09

**Authors:** Anna Christen, Marialuisa Cavelti, Rolf-Dieter Stieglitz, Beatrice Abt-Mörstedt, Hannes Bitto, Patricia E. Newark, Franz Moggi, Kerstin Gabriel Felleiter, Niklaus Stulz, Andreas Riedel, Salvatore Corbisiero

**Affiliations:** 1 Department of Psychology,University of Zurich, Zürich, Switzerland; 2 Lucerne Psychiatry, Lucerne, Switzerland; 3 University Hospital for Child and Adolescent Psychiatry and Psychotherapy, University of Berne, Berne, Switzerland; 4 Department of Psychology, Division of Clinical Psychology and Psychotherapy, University of Basel, Basel, Switzerland; 5 Division of Clinical Psychology and Psychiatry, University of Basel Psychiatric Clinics, Basel, Switzerland; 6 University Hospital of Psychiatry and Psychotherapy, University of Berne, Berne, Switzerland

**Keywords:** ADHD, BPD, emotional dysregulation, psychopathology, self-concept, symptom overlap, impulsivity

## Abstract

**Background:**

Individuals with attention-deficit/hyperactivity disorder (ADHD) and individuals with borderline personality disorder (BPD) show symptomatic overlaps. They both suffer from deficits in emotional regulation, are impulsive and have problems with their self-concept. Therefore, a precise diagnostic differentiation is of great importance. The aim of this study was to find symptom overlaps and differences in patients with ADHD and BPD.

**Methods:**

80 patients with ADHD, 55 patients with BPD and 55 healthy controls were examined regarding their ADHD and BPD symptoms and their degree of emotional dysregulation using self-report instruments.

**Results:**

Patients with ADHD and patients with BPD did not differ significantly in their expression of emotional dysregulation. However, the ADHD patients showed higher scores in impulsivity, inattention, and hyperactivity, whereas the group with BPD showed higher scores in self-concept problems and suicidal behaviour. The two clinical groups showed significantly higher scores in emotional dysregulation and all other symptom domains compared to the control group.

**Conclusion:**

The symptom overlap in emotional dysregulation yields implications for both further research and diagnosis of ADHD. Further studies should define emotional dysregulation consistently to examine the same construct. Key Practitioner Message: This article yields implications that individuals with ADHD and BPD have several symptom overlaps and in fact have no difference in their emotional dysregulation. This has a vast importance for differential diagnosis and treatment of ADHD.

## Introduction

Adults with attention-deficit/hyperactivity disorder (ADHD) have certain symptomatic commonalities with individuals with borderline personality disorder (BPD) (1). To date, research has shown overlaps in the areas of emotional dysregulation and impulsivity (2–6). In addition, there may be an overlap in the self-concept domain, as both disorders show deficits in this area (7–12). In this study, we focused on emotion dysregulation, impulsivity, and self-concept because they represent central constructs in understanding the overlap and distinctions between ADHD and BPD. Impulsivity is strongly associated with ADHD but is also prevalent in BPD, whereas disturbances in self-concept are a hallmark of BPD yet have also been reported in individuals with ADHD. Emotion dysregulation, in turn, has been proposed as a transdiagnostic process that may underlie both disorders and account for shared clinical features. By integrating these constructs, we aimed to clarify whether they reflect common underlying mechanisms or disorder-specific patterns, thereby providing a more nuanced understanding of similarities and differences between ADHD and BPD.

### Emotional dysregulation

Emotional dysregulation represents a key symptom of BPD (13). People with BPD show difficulties in recognizing and labelling emotions, as well as facing and tolerating distress when pursuing their goals. Furthermore, it seems that people with BPD have a lack of appropriate regulation strategies and tend to choose maladaptive regulation strategies (2). Similarly, research shows that ADHD patients exhibit emotional dysregulation and that this is an essential component of the disorder (5, 14–16). More recent research can confirm these findings (17–19). In fact, up to 70% of individuals with ADHD suffer from emotional dysregulation (20). Emotional dysregulation in adults with ADHD is defined by *temper control* (continuous irritability, diminished frustration tolerance, and emotional outbursts), *affective lability* (frequent and short changes from elevated to depressed or agitated moods), and *emotional overreactivity* (frequent outburst and excessive emotional reaction in response to everyday stresses) (21, 22).

There is a heterogeneous body of research on differences in the severity of emotional dysregulation in individuals with ADHD and those with BPD. Certain studies find differences in emotional dysregulation between both patient groups. Rüfenacht et al. (5) found that ADHD patients showed less emotional excitability and intensity of emotions. Their emotions also eased more quickly, compared to those with BPD. Furthermore, participants used more functional strategies to regulate their emotions than the group with BPD (5). The authors suggest that emotional dysregulation, while of significance in ADHD, is a less central component of the disorder than in BPD. However, Cavelti et al. (2) found no significant difference in emotional dysregulation between the two clinical groups. They assessed emotional dysregulation using the *Emotion Regulation Skills Questionnaire* (ERSQ) by Berking & Znoj (23). Witt, Brücher, Biegel, Petermann, & Schmidt (24) found more functional coping strategies for tension in individuals with ADHD, but the more pronounced the ADHD symptomatology, the higher the emotional reactivity and the use of dysfunctional strategies. They also pointed out that individuals with ADHD regulate their emotions through extreme external stimulation, whereas individuals with BPD are more likely to engage in self-injurious behaviours to resolve internal tension and negative feelings. We hypothesize that the heterogeneity in the research findings can be attributed to the use of diverse measures for assessing emotional dysregulation in individuals with ADHD and BPD. Currently, there is no consensus on the definition of emotional dysregulation or on the appropriate instruments for its measurement.

In their meta-analysis Beheshti et al. (14) found that ADHD symptom severity has a strong relationship with emotional dysregulation (*r* = 0.54) and contributes significantly to the differentiation between individuals with or without ADHD. Similarly, Corbisiero et al. (15) and Slobodin et al. (18) found that emotional dysregulation is an indicator of the severity of ADHD symptoms. As emotional dysregulation is an extensive field and to date there is no homogenous definition of the concept, we focused on emotional dysregulation being the sum of emotional impulsivity and emotional lability in this study. This definition stands in accordance with Beheshti et al. (14). We acknowledge that there are numerous measures for assessing emotional dysregulation in ADHD. However, our focus is on the *Emotional Impulsivity Scale* (EIS) (25), which assesses emotional impulsivity, and the *Emotional Lability Scale*, which assesses emotional lability within the Conner’s Adult ADHD Rating Scale (CAARS) (26) framework.

We are aware that emotional dysregulation can be considered to include, but is not limited to, the combined phenomena of emotional lability and emotional impulsivity.

### Impulsivity

Impulsivity is a strongly present factor in both ADHD and BPD (13) however, the brain structures associated with this trait are different. BPD is primarily associated with abnormalities in the prefrontal cortex and limbic system, whereas the alterations seen in ADHD tend to focus on the caudate nucleus and frontostriatal pathways (27). Impulsivity in ADHD is expressed by hasty actions that occur without thinking and on the spur of the moment. Often these actions entail a high risk of harming oneself. Impulsivity also refers to the desire for immediate reward or to the inability to postpone the gratification of one’s needs. According to DSM-5 social intrusions, such as excessive interrupting of others, may also reflect impulsivity in patients with ADHD (13). Impulsive behaviour is manifested in BPD by difficulties suppressing needs, the inability to control outbursts of anger, the tendency to act unexpectedly without considering the consequences, and self-harming behaviour (1). Patients with ADHD were found to show higher levels of impulsivity than patients with BPD (28). However, Witt et al. (24) and Khosravi (29) found no significant difference in impulsivity or impulse control between patients with ADHD and those with BPD. In short, research on the differences in the level of impulsivity in ADHD and BPD is both scarce and heterogeneous. Therefore, the current study will examine if there is a difference between the level of impulsivity in ADHD and BPD.

### Self-Concept

Individuals with ADHD or BPD show deficits in the self-concept (7, 8, 10–12, 30, 31) but it is unclear whether they differ in severity. To date there is no definition on self-concept problems in ADHD per se. However, in this study we focus on the conceptualization of self-concept problems according to Conners et al. (26). Their conceptualization focuses on how ADHD symptoms have led to frustration, academic and social difficulties, and lack of social validation, which is why they assessed self-concept problems in the CAARS. The scale problems with self-concept includes items relating to low self-esteem, self-criticism, and failure to confront challenges (32). Individuals with ADHD often have many negative experiences throughout their lives. This is partly due to their neuropsychological impairments, which are reflected in decreased performance. In particular, interpersonal, academic, and vocational difficulties are common in individuals with ADHD. These negative consequences affect the formation of self-esteem and the self-concept (30). Jones & Hesse (8) provided evidence that stereotypes such as «problem child» or «challenging child» significantly influenced responses after receiving an ADHD diagnosis. For certain individuals, ADHD represents more than just a mental disorder. Society’s view of their inabilities and the unclear separation between their actual limitations and society’s perception of them present another obstacle to develop a stable self-concept. There were also subjects in the study who viewed certain features of the disorder as positive, such as high levels of physical and mental activity (8).

Individuals with BPD experience interpersonal problems on a daily basis (33), and since one’s self-concept is influenced by how one interacts with others these interpersonal problems can negatively shape their self-concept (34). Since individuals with BPD tend to have a fragmented and rather negative self-image (35), this can in turn make them even more vulnerable to interpersonal conflict (36), which can negatively affect their self-concept. According to Van Schie et al. (36), individuals with BPD focus predominantly on negative feedback, leading to the development and maintenance of a negative self-image. Regarding self-concept problems, there is still no research comparing adult ADHD and BPD in terms of this concept.

### Connection between ADHD and BPD

The cause for the named overlapping symptoms could be the high degree of comorbidity between the two disorders. A recent meta-analysis estimated that approximately 21.9% (95% CI: 12.4–33.1) of individuals with ADHD meet criteria for BPD (37) According to a review conducted by Weiner et al. (38) comorbid ADHD in adults with BPD is frequent in clinical settings (16.1–38% of BPD patients). There is an ongoing discussion that ADHD may be a precursor to BPD (39, 40). Adults with BPD retrospectively often report ADHD symptoms in childhood (41–44). Philipsen (45) found a striking convergence of neuropathology in patients with ADHD and BPD, which also suggests an overlapping neurobiology of the two disorders.

According to a more recent study by Pan et al. (46) both ADHD and BPD exhibit hypo-activated abnormalities in the left inferior parietal lobule and altered clusters of the bilateral superior temporal gyrus were disjunctive in ADHD and BPD.

### Differences between ADHD and BPD

Due to the overlapping symptoms, ADHD and BPD must be distinguished well from each other in terms of differential diagnosis. First, the two disorders differ in hyperactivity and inattention. Inattention and hyperactivity are considered to be primary symptoms of ADHD and are more elevated in ADHD than in BPD (4, 47). Furthermore, they differ in terms of the suicidal tendencies and self-harming behaviour, which according to ICD-10 are more pronounced in BPD. Self-harming behaviour was found less frequently in patients with ADHD than in patients with BPD (43). Meta-analytic evidence revealed that a pool rate of suicidal ideation was 80% (95% CI: 61%–94), suicide attempts 52% (95% CI: 47%–58%), and suicides 6% (95% CI: 4%–8%) (48). Suicidality and self-harming behaviour in ADHD are possible consequences of the disorder rather than a defining symptom (45, 49, 50).

The aim of this study was to assess overlaps and differences in ADHD and BPD, since recent research has shown that both ADHD and BPD suffer from emotional dysregulation, impulsivity, and problems with their self-concept but yield heterogeneous findings in emotional dysregulation and impulsivity. No research whatsoever was done comparing adults with ADHD and adults with BPD in self-concept problems. We hypothesized that adults with ADHD and adults with BPD would report the same level of emotional dysregulation (1), impulsivity (2), and problems with their self-concept (3). In contrast, we expected that individuals with ADHD would report higher inattention (4) and hyperactivity levels (5) than individuals with BPD. We additionally expected that adults with BPD would report higher levels of suicidal tendencies than adults with ADHD (6). Finally, we expected that both individuals with ADHD and individuals with BPD report higher levels of overall symptoms, compared to healthy controls (HC) (7).

## Methods

### Participants and study procedure

Participant recruitment took place between 2013 and 2018 in Switzerland (Basel and Bern).

The current study was nested within a randomized controlled trial of treatment for ADHD. General inclusion criterion for all groups (ADHD, BPD, HC) was an age between 18 and 65 years. General exclusion criteria were insufficient command of German, a lifetime diagnosis of schizophrenia or another psychotic disorder, and a current or recent episode of mania, severe major depression, acute stress disorder or substance dependence as well as an IQ below 85. Recruitment efforts resulted in 89 participants with ADHD from the ADHD Special Consultations Unit of the Outpatient Department, University of Basel Psychiatric Clinics. Eligible participants met the criteria of the ICD-10 (51) ADHD diagnosis. Two independent experts confirmed patients’ diagnosis of ADHD based on clinical interviews, self-rating and observer-rating scales, developmental and psychiatric history, school certificates and/or reports from former teachers, if available. The diagnoses were made in accordance with current guidelines (National Institute for Health and Clinical Excellence (NICE), n.d) and the following diagnostic instruments were used: *The Wender-Reimherr Interview* (WRI) (52), the *Wender Utah Rating Scale* (WURS-k) (53) and the *Conners Adult ADHD Rating Scale* (CAARS-S) (54).

We recruited 55 participants with BPD according to ICD-10 (51). They had received treatment as usual at the University of Basel Psychiatric Clinics, or the University Hospital of Psychiatry and Psychotherapy Berne. Diagnoses were rendered by trained clinical psychologists and psychiatrists. As general procedure differential diagnoses were assessed by the clinicians. Patients with BPD and comorbid ADHD were excluded from this study.

The 55 control participants were employees of the University of Basel Psychiatric Clinics or students from the University of Basel. HC were matched in terms of age and gender to the ADHD group.

A member of the study team informed all participants about the aims, risks, benefits, and procedure of the study, and received their written informed consent and this study was performed in accordance with the *Declaration of Helsinki*. The participants then underwent an assessment of about 45 to 60 min to answer a set of self-report measures. The study was approved by local ethics committees (Ethics Committee of Basel [Ethikkommission Nordwest- und Zentralschweiz], P2BSP1_165354).

### Measures

The German version of the *Conners Adult ADHD Self Rating Scale* (CAARS-S) (54) was used to assess the current severity of ADHD symptoms. This self-report measure includes 66 items that are rated on a 4-point Likert-scale (0 = not at all, 3 = very much) and is divided into nine subscales: *Inattention/Memory Problems*, *Hyperactivity/Restlessness*, *Impulsivity/Emotional Lability* and *Self-Concept Problems* as well, *ADHD Index* that contains items designed to differentiate between individuals with and without ADHD. The total score of the individual subscales is formed by the sum of the items, with higher scores indicating higher symptom severity (26). The subscale *Impulsivity/Emotional Lability* was divided into two separate subscales in this study. In the current study, internal consistencies of the subscales were excellent, with Cronbach’s alpha = .87 to .88.

To assess emotional impulsivity the *Emotional Impulsivity Scale* (EIS) (25) was filled out by the participants. The EIS includes seven items that are rated on a 4-point Likert-Scale (0 = never, 3 = very often) over the last six months. The total score of the scale is formed by the sum of the seven items, with a high score indicating higher emotional impulsivity. In the current study Cronbach’s alpha was α = .91, which indicates excellent internal consistency. The EIS was translated into German by Corbisiero, Mörstedt, and Stieglitz. This measure has not yet undergone a formal validation study.

To assess current BPD symptoms, the German short version of the *Borderline Symptom List* (BSL-23) (55) was used. The BSL-23 includes 23 items, which are rated on a 5-point Likert-scale (0 = not at all, 4 = very strong) on their occurrence within the last seven days. The total score is calculated by averaging items. Higher scores indicate higher symptom severity (55). The internal consistency in the current study was excellent, with Cronbach’s alpha = .96. To assess current suicidal tendencies, the corresponding items of the BSL-23 were conflated into a separate subscale. Cronbach’s alpha was α = .85, indicating excellent internal consistency.

### Statistical analyses

Data analysis was performed using the statistical program IBM SPSS Statistics (version 26), as it was the current institutional standard at the time of analysis (2020).

Of the 198 participants, eight were excluded from data analysis because they were diagnosed with both ADHD and BPD, resulting in N = 190. Furthermore, one participant with BPD did not complete the CAARS-S, resulting in a smaller sample size for the subscales of the CAARS-S.

To test for normal distribution the Q-Q diagram and the histogram were used. In addition, the scales were checked for their variance homogeneity, using the Levene test. To analyse the group differences between the two patients and the control group a Kruskal Wallis test was performed. We subsequently conducted Dunn–Bonferroni-adjusted pairwise comparisons to evaluate specific between-group differences. *t*-tests were used to test for overlaps and differences between the group with ADHD and the one with BPD. The Bonferroni Correction was used to control for alpha error inflation. Since the scale *Suicidal Behaviour* of the BSL-23 did not meet the criteria for a parametric test, a Mann-Whitney U test was performed.

## Results

The demographic and clinical characteristics of this study sample are shown in **Table 1**. In total 190 participants were examined. All clinical groups differed significantly in gender and years of education, but not in age. The mean age was 32.42 years (*SD* = 10.35). Almost all participants with BPD were female. In the other two groups, the majority of the participants were male. Controls reported more years of education than the individuals with ADHD. ADHD participants had more years of education than individuals with BPD. Furthermore, **Table 1** compares all groups in terms of their symptom severity. It displays that HC showed significantly lower scores in all assessed symptoms. Differences between ADHD and BPD are shown in more detail in **Table 2**.

**Table 1:** Demographic and clinical variables.

	ADHD	BPD	HC	Group comparison	Pairwise comparison
Scale	*M*(*SD*)/*n*(%)	*M*(*SD*)/*n*(%)	*M*(*SD*)/*n*(%)		
Gender (f/m)	30/50 (37.5/62.5%)	52/2 (96.4/3.6%)	25/29 (45.5/54.5%)	χ2(2) =50.13***	
Age	33.39 (9.40)	30.11 (10.24)	33.33 (11.56)	*F*(2, 187) = 1.95	
Years of education	11.75 (2.23)	10.35 (2.70)	13.63 (3.26)	Welchs *F*(2, 103.55) = 16.05***	
	Rank	Rank	Rank	Kruskal Wallis H	
CAARS -S Inattention/Memory Problems	130.69	100.09	38.08	94.18***	ADHD > BPD > HC
CAARS-S Hyperactivity/Restlessness	128.94	97.06	43.61	79.52***	ADHD > BPD > HC
CAARS-S Impulsivity	130.10	95.61	43.35	82.42***	ADHD > BPD > HC
CAARS-S Emotional Lability	118.90	117.49	38.15	84.17***	ADHD = BPD > HC
CAARS-S Problems Self- Concept	105.03	135.39	40.76	86.47***	BPD > ADHD > HC
CAARS-S ADHD Index	129.47	105.78	31.42	108.12***	ADHD > BPD > HC
EIS	123.72	116.75	33.2	100.190***	ADHD = BPD > HC
BSL-23	105.38	141.26	34.49	108.86***	BPD > ADHD > HC
BSL-23 Suicidal Behaviour	93.27	136.75	56.53	71.91***	BPD > ADHD > HC

ADHD, attention-deficit/hyperactivity disorder; BPD, borderline personality disorder; HC, healthy controls; EIS, Emotional Impulsivity Scale; CAARS-S, Conners’ Adult ADHD Rating Scale-Self-Report; BSL-23, Borderline Symptom List-Short Version.

***p <.001.

**Table 2:** Effects of the group differences between ADHD and BPD.

	ADHD (*n* = 80)	BPD (*n* = 55)	Group differences	
Scale	*M* (*SD*)	*M* (*SD*)	*t*(df)	*d*
CAARS-S Inattention/Memory	22.23 (6.79)	17.37 (6.15) (*n* = 54)	4.22 (132)***	.35
CAARS-S: Hyperactivity/Restlessness	20.77 (7.18)	15.57 (8.22) (*n* = 54)	3.88 (132)***	32
CAARS-S: Impulsivity	9.92 (3.56)	7.30 (4.09) (*n* = 54)	3.94 (132)***	.32
CAARS-S: Emotional Lability	10.89 (3.89)	10.70 (3.59) (*n* = 54)	.277 (132)	.02
CAARS-S: Problems with Self- Concept	10.30 (4.32)	13.13 (3.81) (*n* = 54)	-3.90 (132)***	.32
EIS	13.96 (4.36)	13.31 (3.90)	.89 (133)	.07
	Rank	Rank	*U (*Z)	*r*
BSL-23: Suicidal Behaviour	53.49	88.25	1039 (-5.27)***	.46

ADHD, attention-deficit/hyperactivity disorder; BPD, borderline personality disorder; HC, healthy controls; EIS, Emotional Impulsivity Scale; CAARS-S, Conners’ Adult ADHD Rating Scale-Self-Report; BSL-23, Borderline Symptom List-Short Version.

***p < 0.01.

The BPD group and the ADHD group did not differ significantly in their scores on the EIS (see **Table 2**). Furthermore, the two groups did not differ significantly in *Emotional Lability* of the CAARS-S. Contrary to our hypotheses, the t-test showed that the two patient groups were significantly different in terms of *Impulsivity* as measured by the CAARS-S. Patients with ADHD showed higher expressions than patients with BPD. In addition, the two groups differed significantly on *Problems with Self-Concept* measured by the CAARS-S. Patients with BPD showed a higher expression in the scale *Problems with Self-Concept* than patients with ADHD. The data analysis showed that the clinical groups differed significantly in the symptoms of *Inattention/Memory Problems* and *Hyperactivity/Restlessness*. Patients with ADHD showed a higher expression in the scale *Inattention/Memory problems* than the group with BPD. Individuals with ADHD showed a significantly higher expression in the scale *Hyperactivity/Restlessness* than individuals with BPD. Individuals with BPD showed higher expression in the *Suicidal Behaviour* items of the BSL-23 than individuals with ADHD.

## Discussion

The aim of this study was to assess symptom overlaps and differences between patients with ADHD and patients with BPD. Data analysis showed no significant difference in emotional dysregulation between patients with BPD and patients with ADHD. The group with ADHD had higher scores in *Hyperactivity/Restlessness*, *Inattention/Memory Problems*, and *Impulsivity*. In contrast patients with BPD had higher scores in *Problems with Self-Concept* and *Suicidal Behaviour*.

Both clinical groups differed significantly from the healthy control group in all scales. Patients with BPD also showed higher scores in the scale’s *Inattention/Memory Problems* and *Hyperactivity/Restlessness* than the control group, even though these symptoms are not associated with BPD. Accordingly, it could be assumed that individuals with BPD are less inattentive, hyperactive, and impulsive than individuals with ADHD, yet they are more inattentive, hyperactive, and impulsive than a control group. Patients with ADHD showed less suicidal behaviour and fewer self-concept problems than those with BPD, but they scored higher on *Suicidal Behaviour* and *Problems with Self-Concept* than individuals from the control group.

### Symptom overlaps between ADHD and BPD

The first aim of this study was to find overlaps in the symptomatology of ADHD and BPD.

Patients with ADHD did not differ significantly from those with BPD in their severity of emotional dysregulation. This result is inconsistent with the findings of Rüfenacht et al. (5), which found better emotional regulation in patients with ADHD. One reason for the different results could be the instruments used to assess emotional regulation of the participants. Rüfenacht et al. (5) used *the Emotion Reactivity Scale* (ERS; (56) and the *Cognitive Emotional Regulation Questionnaire* (CERQ; (57). The ERS is a self-report instrument that examines subject’s emotional reactivity. The CERQ assesses the adaptive and maladaptive strategies that the subjects use to regulate their emotions. Furthermore, Kenézlői et al. (4) found that individuals with BPD scored higher on emotional dysregulation using the *Difficulties in Emotion Regulation Scale* (DERS) (58). In the current study, regulation strategies were not explicitly collected. This could be a reason for the heterogeneous results. However, the analyses of the current study overlap with the study by Cavelti et al. (2), examining participants by using the *Emotion Regulation Skills Questionnaire* developed by Berking & Znoj (23). The findings can be highlighted by another study that concluded that individuals with ADHD or BPD do on average not differ in how they experience and express anger, nor do they differentiate in their aggressive behaviours and substance abuse. All these behaviours are indicators of a lack of emotional regulation (28). Furthermore, Moukhtarian et al. (59) found no difference in emotional dysregulation comparing women with ADHD or BPD and conclude that emotional dysregulation is unlikely to be helpful in distinguishing between ADHD and BPD. To sum up, the current findings on emotional dysregulation remain unclear. The reason for the heterogeneous evidence could be the ambiguous definition of emotional dysregulation and thus the different operationalization of the construct.

Contrary to the postulated hypothesis, it turned out that the patients with ADHD showed a higher expression in the subscale *Impulsivity* of the CAARS-S. This is consistent with the study by Prada et al. (28) and Kenézlői et al. (4) who also found that patients with ADHD had higher symptom expression in impulsivity. On the other hand, it contradicts the null results of Witt et al. (24) and Khosravi (29). The reason for this could again be the inconsistent definition and measurement of impulsivity.

In addition, the data analysis was inconsistent with the hypothesis that problems with self-concept would not result in a significant difference between the patient groups. The group with BPD had a significantly higher score on the *Problems with Self-Concept* subscale of the CAARS-S. This, however, confirms the hypothesis of Sheehan, Nieweglowski, & Corrigan (60) that affected individuals with BPD may have elevated scores in problems with self-concept, compared to individuals with ADHD. They found increased initialization of stigma in patients with BPD, which in turn could weaken their self-concept.

### Differences between ADHD and BPS

The second aim was to examine the symptoms that distinguish ADHD and BPD. The patients with ADHD were found to have higher scores on the scales *Inattention/Memory Problems* and *Hyperactivity/Restlessness*, compared to the patients with BPD. The examination of suicidal behaviour also confirmed the formulated hypothesis that suicidal behaviour is significantly more pronounced in patients with BPD than in patients with ADHD. Suicidal behaviour was assessed using three items from the BSL-23, which were used to describe suicidal behaviour. This limits the exact assessment and could therefore influence the reliability of the results.

### Symptom severity in clinical and control group

The third aim was to assess whether the control group showed less general symptom severity, which was proved consistent with hypothesis (7). This finding seems not to be surprising at first glance; nevertheless, it is noteworthy that patients with ADHD differed significantly from the control group in the BSL-23, as do patients with BPD on all ADHD symptoms. This means that individuals with ADHD have a certain amount of BPD symptoms. For example, the pairwise comparison between the group with BPD and the control group showed a significant difference in the scales *Hyperactivity/Restlessness* and *Inattention/Memory Problems* with effect sizes according to Cohen (61) between *r* = .49 and .56. Nevertheless, the high scores in the scale *Inattention/Memory Problems* in patients with BPD could also be due to dissociative characteristics, which were not assessed in this study, but in a study examining 103 participants with ADHD or BPD, the symptom hyperactivity did not discriminate between the two groups (62). Although, in the current study, the two clinical groups differed in the symptom hyperactivity, in practice, depending on the questionnaire and the severity of a patient’s symptoms, this difference might not be sufficiently clear, and it could lead to difficulties in differential diagnosis (6). The mentioned results can support Philipsen’s (45) assumption – discussed in the introduction - that the two disorders could be two dimensions of one disorder. Which is also supported by more recent research (40).

### Limitations

There are several limitations in this study, which should be considered in further studies and should be taken into account when interpreting the results. One limitation is the gender distribution of the sample. In the BPD group, the rate of male participants was only 2%; in the ADHD group, the distribution was 37.5% females and 62.5% males. The control group had a ratio of 45.5% female to 54.5% male participants. Given that emotion regulation strategies are known to differ between men and women—women experiencing negative emotions more intensely than positive emotions (63) and more often employing internalizing strategies such as rumination. While men more often use externalizing strategies such as alcohol misuse (64).

Furthermore, the control group was not matched in terms of demographic and socio-economic variables. Full matching on additional variables than age and gender is often not feasible and may introduce selection bias or reduce sample representativeness (65, 66). Residual confounding due to unmeasured or incompletely matched variables is therefore an inherent limitation of observational designs. In future research, larger samples should allow for control of gender and education variables through covariate analysis or matching. Furthermore, the use of psychopharmacological drugs could bias the results. Among patients with BPD, 78.2% were taking a psychopharmacological medication. In contrast, 40% of patients with ADHD were taking a psychopharmacological medication. In addition, the specific medication was not collected in the assessment.

In the assessment of emotional dysregulation, the aspect of emotional impulsivity and the aspect of emotional lability were assessed. However, emotional dysregulation includes various facets such as being able to recognize and label emotions, being able to identify triggers for the emotional reaction, as well as being able to correctly interpret emotion-based physiological responses (23). Please also note, that the EIS (67) has not undergone a formal validation study yet. As a result, it is possible that although the two clinical groups did not differ significantly in the areas of emotional impulsivity and emotional lability, the effect found would not emerge in a broader assessment of emotional dysregulation. It is important to point out that assessment of emotional lability, impulsivity and problems with self-concept was conducted using a questionnaire constructed for assessing ADHD symptoms. This could limit the proper assessment of the symptoms in individuals with BPD. This issue applies to all the domains assessed with the CAARS-S, since the scales are designed to assess individuals with ADHD and not BPD. A further limitation is the reliance on self-report measures for both ADHD and BPD symptom assessment. Self-report measures show a high false-positive rate in ADHD. However, ADHD screening measures have excellent ability to identify non-ADHD individuals correctly (68). The agreement between self-report and clinical interviews is moderate and often higher in BPD (69).

### Implications

This study provides the first research on self-concept problems comparing individuals with ADHD and BPD. Though Sheehan et al. (60) examined the internalization of stigma in the two clinical groups, no actual research on self-concept problems has been conducted to date. Further studies should examine in more detail the self-concept problems that affect both patient groups. Furthermore, future studies should address the relationship between ADHD and other personality disorders. ADHD is also known to be related to antisocial and narcissistic personality disorder (70). Longitudinal studies are important in examining the association between ADHD and personality disorders to understand the aetiology. Both risk and protective factors, such as attachment style, intelligence, social support, etc., should be collected to better understand the course from ADHD to a personality disorder.

It seems that the three main symptoms cannot explain the complete spectrum of ADHD (Corbisiero, Riecher-Rössler, et al., 2017). According to the results of the current data analysis emotional dysregulation represents an essential aspect of ADHD and could be as crucial as the symptoms of inattention, hyperactivity, and impulsivity (71). Although emotional dysregulation is a leading symptom of BPD, it seems to have the same expression in ADHD according to the current study. In the ICD-10 and the DSM-5 however, it is only referred to and despite the findings regarding the high level of emotional dysregulation, it is not considered a characteristic symptom in the diagnostic criteria of ADHD. Consequently, emotional dysregulation should be assessed separately in the diagnostic assessment of ADHD in order to obtain information about the course, symptom expression, and possible comorbid disorders.

Future research should examine emotion dysregulation in ADHD and BPD using larger and more gender-balanced samples, ideally with longitudinal designs to clarify potential developmental links between the two disorders. Multivariate approaches could help disentangle the contributions of related constructs such as impulsivity and self-concept. Moreover, future studies should address gender-specific regulation strategies and employ instruments that capture both internalizing and externalizing forms of emotion regulation to better reflect the complexity of these processes. Furthermore, when examining emotional dysregulation, the construct should be defined consistently and a general instrument to assess emotional dysregulation should be used. This also applies to the assessment of impulsivity and problems with the self-concept. Further studies should unify this deficit and use a consistent operationalization.

## Conclusion

ADHD and BPD exhibit overlapping symptom profiles, particularly with regard to emotional dysregulation, which did not differ significantly between the two groups in the present study. However, distinct patterns emerged in other domains: impulsivity was more pronounced in ADHD, whereas disturbances in self-concept were somewhat more elevated in BPD. In addition, the disorders differed in levels of inattention and hyperactivity, as well as in the presence of suicidal tendencies. Overall, these findings highlight both shared and disorder-specific features, underscoring the importance of careful differential diagnosis. They further suggest that assessment and treatment approaches should account for these overlapping and distinct characteristics to better tailor interventions to the needs of individuals with ADHD and BPD.

## Data Availability

The raw data supporting the conclusions of this article will be made available by the authors, without undue reservation.
